# Contributions of Reduced Susceptibility Alleles in Breeding Apple Cultivars with Durable Resistance to Fire Blight

**DOI:** 10.3390/plants10020409

**Published:** 2021-02-22

**Authors:** Sarah A. Kostick, Soon Li Teh, Kate M. Evans

**Affiliations:** 1 Department of Horticulture, Washington State University, Tree Fruit Research and Extension Center, Wenatchee, WA 98801, USA or kosti028@umn.edu (S.A.K.); soonli.teh@wsu.edu (S.L.T.); 2 Department of Horticultural Science, University of Minnesota, Saint Paul, MN 55108, USA

**Keywords:** *Erwinia amylovora*, *Malus domestica* Borkh, durable host resistance, DNA-informed breeding, rapid cycle breeding

## Abstract

Breeding apple cultivars with durable genetic resistance is a potential long-term solution to fire blight, a devastating bacterial disease caused by *Erwinia amylovora*. However, phenotyping resistance/susceptibility to fire blight is challenging due to *E. amylovora* strain virulence, differential host × strain interactions, quantitative host resistance, environmental influences on disease, and impacts of tree vigor on susceptibility. Inheritance of resistance/susceptibility to fire blight is complex and phenotypic information alone is insufficient to guide breeding decisions targeting resistance. Several quantitative trait loci (QTLs) associated with resistance/susceptibility to fire blight have been detected throughout the apple genome. Most resistance alleles at fire blight QTLs have been identified in wild *Malus* germplasm with poor fruit quality, which limits their breeding utility. Several QTLs have been identified in populations derived from cultivars and reduced-susceptibility alleles have been characterized in multiple important breeding parents. Although resistance to fire blight is an attractive target for DNA-informed breeding, relatively few trait-predictive DNA tests for breeding relevant fire blight QTLs are available. Here we discuss (1) considerations and challenges associated with phenotyping resistance/susceptibility to fire blight; (2) sources of resistance that have been identified for use as parents; and (3) our perspective on short and long-term strategies to breed apple cultivars with durable resistance to fire blight with emphasis on the potential contributions of reduced susceptibility alleles to achieve this goal.

## 1. Clonally Propagated Apple Orchards Are Vulnerable to Fire Blight Epidemics

**Figure 1:**
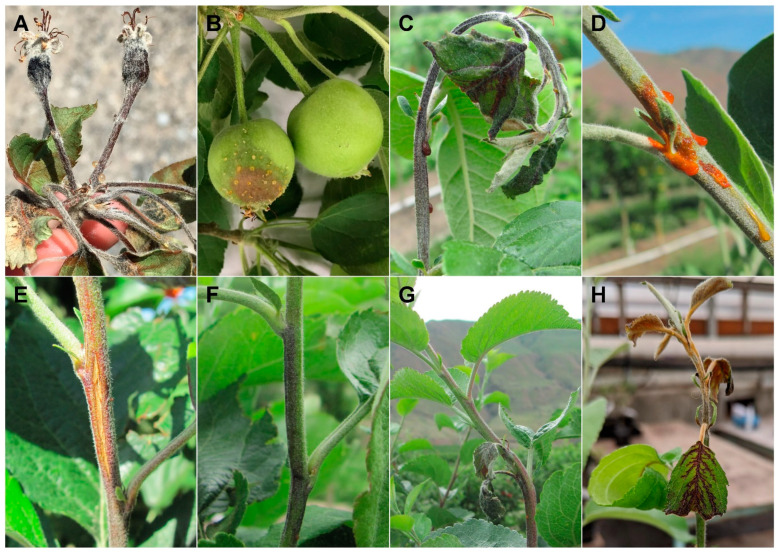
*Erwinia amylovora* can infect the flowers (**A**), fruits (**B**), and vegetative tissue (**C**–**H**) of apple, which can result in bacterial ooze (**B**–**D**) and necrosis (**A**–**H**). A necrotic shepherd’s crook (**C**) is characteristic of a highly susceptible response to invasion of host shoot tissues by *E. amylovora*. Fire blight symptom severity varies among *Malus* cultivars and species. Susceptible responses that are depicted were the result of natural infection of flowers and fruit (**A**,**B**) under field conditions or artificial inoculation of shoots with *Ea* 153n under field (**C**–**G**) or greenhouse (**H**) conditions.

Breeding for resistance offers a potential long-term solution to fire blight, a devastating bacterial disease in apple (*Malus domestica* Borkh) caused by *Erwinia amylovora*. The bacterium, which infects the flowers, fruits, shoots, and rootstock of the tree, can cause severe structural damage and tree death (Figure 1) [1,2]. Fire blight, which has been reported in over 40 countries [3], can result in significant economic costs. For example, in 2018, severe fire blight infections in Washington State, where approximately 66% of the U.S. apple crop is produced [4], resulted in estimated direct costs of over $37 million from chemical sprays, tree removal, and tree replacement [5]. In recent decades, U.S. commercial apple production systems have become more vulnerable to fire blight epidemics due to production of highly susceptible apple cultivars (e.g., Gala, Fuji), the shift towards high-density planting systems, and lack of sustainable control methods that are effective against all disease phases [2]. Breeding resistant apple cultivars could complement current unsustainable control methods (e.g., antibiotics), as discussed in several book chapters and reviews [6,7,8,9]. In a review paper, Emeriewen et al. [10] noted that reduced susceptibility mechanisms for defense against *E. amylovora* in apple cultivars (e.g., ‘Fiesta’) might slow the effects of pathogen mutations and thus, contribute to durable host resistance. Targeting reduced susceptibility (i.e., incomplete or quantitative resistance) might be an effective breeding approach. In this perspective paper, we focus on reduced susceptibility to fire blight in apple and discuss (1) considerations and challenges associated with phenotyping resistance/susceptibility to fire blight; (2) resistance sources that have been identified for use as breeding parents; and (3) short- and long-term strategies for developing apple cultivars with durable resistance to fire blight specifically emphasizing the contributions of reduced susceptibility alleles to achieve this goal.

## 2. Phenotyping Resistance/Susceptibility to Fire Blight

### 2.1. Challenges Associated with Phenotyping Resistance/Susceptibility to Fire Blight

Fire blight incidence and severity are strongly influenced by environmental conditions (e.g., temperature, humidity, and precipitation), host factors (e.g., tree vigor), *E. amylovora* strain virulence, differential host × strain interactions, and quantitative host resistance, making it challenging to phenotype resistance/susceptibility to fire blight [1,9,11]. Different phenotyping methods can provide variable and often uncorrelated results [3]. Phenotyping methods vary by target tissue (e.g., floral and vegetative), inoculation methodology (if any), *E. amylovora* strain, inoculum concentration, evaluation environment (e.g., greenhouse and field), and scoring methodology [3]. Generally, studies have relied on artificial shoot inoculation under greenhouse conditions, e.g., [12,13,14,15,16]. As environmental conditions are more easily controlled in a greenhouse compared to a field environment, greenhouse evaluation increases chances of infection and enables effective identification of highly susceptible individuals and possibly highly resistant individuals [3]. Potted trees in the greenhouse typically perform differently (e.g., vigor) than orchard trees resulting in an overestimation of susceptibility that is not necessarily predictive of field performance, e.g., [17]. Harshman et al. [18] observed low to no correlations (*R*^2^ ranged from 0.0013 to 0.1979) between greenhouse and field evaluation of 121 *M. sieversii* accessions. Harshman et al. [18] reported that most accessions demonstrated similar resistance levels in both greenhouse and field environments even though the *R*^2^ values were low. Fire blight is a quarantine disease in many countries; thus, many research programs are limited to phenotyping resistance/susceptibility to fire blight under controlled greenhouse conditions.

### 2.2. Phenotyping for Selection Versus Identification of Resistance Sources

In a breeding program, phenotyping for resistance/susceptibility to fire blight often occurs at the unreplicated seedling stage in the greenhouse and might result in a terminal selection decision (i.e., cull). For example, in the Washington State University apple breeding program (WABP), seedlings derived from crosses targeting resistance to fire blight are inoculated under greenhouse conditions with an *E. amylovora* inoculum suspension. Seedlings that survive inoculation are planted in the field for reinoculation and further evaluation before being vegetatively propagated.

Phenotyping for identification of resistance sources or quantitative trait locus (QTL) mapping studies requires robust quantitative phenotypic data from standardized phenotyping protocols, evaluation of multiple biological replicates in multiple environments/years, and detailed measurements for each biological replicate (e.g., lesion lengths, incidence, and age of wood infected).Various methodologies for scoring fire blight shoot infection severity have been reported including lesion length, e.g., [13,14], proportion of shoot length blighted (SLB) or proportion lesion length, e.g., [13,14,16,18,19,20,21,22], age of wood infected, e.g., [18,22], and area under the disease progression curve (AUDPC) [14]. In QTL mapping studies, use of various scoring methodologies have resulted in detection of similar QTL. For example, Khan et al. [14] detected the same QTL with log transformed lesion length data, proportion lesion length data at multiple time points after inoculation, and AUDPC data. Differential responses to *E. amylovora* strains in *Malus* have been reported, e.g., [23,24]. For example, Norelli et al. [23] reported that severity of fire blight symptoms on ‘Delicious’ depended on the *E. amylovora* strain (ranged from 0.09 to 0.91 SLB). In a more recent study, Khan et al. [24] demonstrated that susceptibility levels of cultivars Gala, Golden Delicious, and Empire varied depending on the strain (Ea273, E2002A, and E4001A) or combination of strains. Differential responses of *Malus* cultivars to *E. amylovora* strains indicate that resistance sources or QTL identified should be validated with different *E. amylovora* strains.

## 3. Variation for Resistance/Susceptibility among *Malus* Cultivars and Species

### 3.1. Most Commercial Apple Cultivars Are Susceptible to Fire Blight

Identification of resistance sources with high fruit quality (i.e., elite) for use as breeding parents is an important precursor to developing breeding populations with low susceptibility to fire blight assuming moderate to high trait heritability. Most modern commercial apple cultivars are susceptible to fire blight [1,22,25,26,27]. Kostick et al. [22] recently provided an updated comparison of resistance/susceptibility levels of 94 important breeding parents (IBPs) and cultivars. Similar to previous studies, e.g., [25,26,27], most apple cultivars (e.g., Jonathan, Ginger Gold, Sansa, and Sweet Sixteen) demonstrated high to moderate susceptibility to fire blight [22]. Several moderate to highly resistant cultivars were confirmed, with eight cultivars (i.e., Dolgo, Enterprise, Frostbite, Kidd’s Orange Red, Tsugaru, Vista Bella, Wildung, and Williams’ Pride) being classified as highly resistant in one year and moderately resistant in the other [22].

### 3.2. Wild Malus Species as Sources of Resistance to Fire Blight

Most sources of resistance to fire blight (donors) have been characterized in diverse *Malus* germplasm with poor fruit quality. Over a 10-year period, Forsline and Aldwinckle [28] recorded natural occurrence and severity of fire blight infections among the 2351 *Malus* accessions in the USDA-ARS Plant Genetic Resources Unit (PGRU). Forsline and Aldwinckle [28] observed that 46% (n = 1091) of accessions were consistently infected at high severity whereas 25% of accessions (n = 596) did not exhibit symptoms under relatively high fire blight pressure. Examples of accessions that did not exhibit fire blight symptoms or had minor infections included Budagovsky 491, *Malus orientalis*, *Malus robusta* 5, and Malling 7 [28]. More recently, Khan and Chao [29] analyzed field observation data, downloaded from the Germplasm Resources Information Network (GRIN-Global database), for shoot blight of 2318 accessions from 33 *Malus* species and blossom blight of 638 accessions from 14 *Malus* species in the USDA-ARS PGRU *Malus* germplasm collection. More than 60% of accessions of several species including *Malus ombrophila*, *Malus prattii*, *Malus fusca*, *Malus sieversii*, and *Malus halliana* were classified as resistant to fire blight shoot infections whereas approximately 75% of *M. domestica* accessions were highly susceptible [29]. Additionally, more than 60% of accessions from several species (e.g., *Malus angustifolia*, *Malus ioensis*, and *M. sieversii*) had resistant scores for fire blight blossom infections [29]. Harshman et al. [18] examined resistance/susceptibility to fire blight of approximately 200 *M. sieversii* accessions and identified 12 accessions that were as resistant as the resistant controls (*M. robusta* 5, ‘Delicious’). These studies demonstrate that there is variation within and among *Malus* species for resistance/susceptibility to fire blight.

## 4. Quantitative Phenotypic Variation for Resistance/Susceptibility to Fire Blight within and among Offspring in Families

Phenotypic variation for resistance/susceptibility to fire blight in segregating populations has been examined after natural infection in the field, e.g., [30] or artificial inoculation under greenhouse or field conditions, e.g., [15,17,31,32]. Many of these studies have reported quantitative phenotypic variation for resistance/susceptibility to fire blight in various segregating populations. Kostick et al. [33] examined phenotypic variation for resistance/susceptibility levels over two years among and within 32 full-sib families (n = 314 offspring) that represented 27 IBPs of a pedigree-connected apple breeding germplasm set. Offspring responses, quantified as adjusted shoot length blighted best linear unbiased predictions (SLB BLUPs), ranged from highly resistant to highly susceptible (0.04–0.97 across years) [33]. Kostick et al. [33] reported that across years approximately 18%, 37%, and 44% of offspring had low (≤0.25 SLB BLUPs), moderate (0.25 to ≤0.50 SLB BLUPs), and moderately to highly susceptible (>0.50 SLB BLUPs) responses, respectively. Quantitative phenotypic variation for resistance/susceptibility to fire blight has been observed in families derived from susceptible × susceptible crosses [15,17,33]. For example, Kostick et al. [33] reported variation within multiple full-sib families derived from two susceptible parents including a ‘Braeburn’ × ‘Ginger Gold’ family with 24 offspring (0.12–0.87 SLB BLUPs across years) and a ‘Sansa’ × ‘Granny Smith’ family with 11 offspring (0.26–0.94 SLB BLUPs across years). Quantitative phenotypic variation in susceptible × susceptible families for resistance/susceptibility indicates that susceptible IBPs might be sources of reduced-susceptibility alleles at one or more fire blight QTLs. Therefore, resistance/susceptibility levels of parents might not be predictive of offspring performance, and phenotypic information alone is likely inadequate to guide breeding decisions.

## 5. Moderate Heritability Estimates Indicate Breeders Could Increase Resistance via Selection

Broad-sense heritability, in general, is defined as the proportion of phenotypic variance that is attributed to genetic effects, while narrow-sense heritability is the proportion of phenotypic variance that is explained by additive genetic variance [34]. Luby et al. [30] reported that narrow-sense heritability estimates for resistance/susceptibility to fire blight in a diverse apple germplasm set ranged from 0.05 to 0.85 (most estimates ranged from 0.12 to 0.36) depending on the population. Kumar et al. [35] reported that average narrow-sense heritability estimates for various breeding germplasm populations ranged from 0.27 to 0.38. In a recent study, Kostick et al. [33] estimated variance components and heritabilities for resistance/susceptibility to fire blight in a pedigree-connected apple breeding germplasm set using animal (i.e., individual) models fit to shoot length blighted (SLB) data [33]. Kostick et al. [33] reported broad-sense heritability estimates ranged from 0.44 to 0.46 across years while narrow-sense heritability estimates ranged from 0.22 to 0.49 across models and years. This large range in narrow-sense heritability estimates was likely due to differences among models used. Moderate broad-sense and narrow-sense heritability estimates indicate that breeders can increase resistance to fire blight in breeding germplasm via selection.

## 6. Multiple Additive and/or Epistatic QTLs Associated with Resistance/Susceptibility to Fire Blight Have Been Identified throughout the Apple Genome

### 6.1. Most Fire Blight QTLs Have Been Detected in Wild Malus Germplasm with Poor Fruit Quality

Multiple additive and/or epistatic QTLs associated with resistance/susceptibility to fire blight have been detected throughout the apple genome [12,13,14,16,19,20,21,36,37,38,39,40,41,42,43]. Several large-effect QTLs explaining ≥ 40% of phenotypic variation (PVE) have been characterized in populations derived from wild *Malus* germplasm (e.g., ‘Evereste’, *Malus* *×* *arnoldiana*, *Malus floribunda* 821, *M. fusca*, and *M. robusta* 5) with astringent, crabapple-type fruit [13,19,21,37,38,40]. A large-effect QTL on Chromosome (Chr.) 3 (67–83% PVE) was detected in multiple populations derived from crosses with *M. robusta* 5 and resistance (*R*) gene *FB_MR5*, which is a CC-NBS-LRR (coiled coil domain—nucleotide-binding site—leucine rich repeat) gene, was determined to underly this QTL [19,38,40,44,45]. To date, all candidate fire blight *R* genes were identified in wild *Malus* germplasm [44,45,46,47].

Introgression of resistance alleles from wild sources is possible; however, the long generation times, gametophytic self-incompatibility and high heterozygosity of *Malus* germplasm make improving fruit quality while maintaining resistance challenging [9,48]. Additionally, single sources of resistance are often not durable in perennial production systems because pathogen populations can evolve to overcome specific host *R* genes [8]. For example, the gene on Chr. 3 that underlies the resistance of *M. robusta* 5 was overcome by virulent *E. amylovora* strains [41,49]. Pyramiding of multiple resistance alleles, which decreases the chance of pathogen mutations overcoming host resistance [50], is likely needed to achieve durable resistance to fire blight in perennial apple production systems.

Rapid cycle breeding techniques have been used to accelerate introgression and pyramiding of favorable alleles in apple [51,52,53,54,55,56,57]. Rapid cycle (or fast-track) breeding techniques rely on transgenic intermediate generations that over-express the early flowering gene *BpMADS*4 from silver birch (*Betula pendula*), which shortens the juvenility period, enabling rapid introgression of favorable alleles [52,56]. After several generations, non-transgenic individuals that have inherited the favorable alleles are selected [56].

### 6.2. Fire Blight QTLs Detected in Populations Derived from Apple Cultivars

Several fire blight QTLs have been detected throughout the apple genome (e.g., Chromosomes 2, 3, 5, 6, 7, 8, 9, 10, 12, and 13) in populations derived from cultivars, with most QTLs explaining ≤ 20% phenotypic variation [12,14,16,20,39,43]. A large-effect QTL on Chr. 7 (30–47% PVE) was originally detected in mapping populations derived from crosses with ‘Fiesta’ and later in a population derived from ‘Enterprise’ [12,14,16]. In the 2018 study, van de Weg et al. [16] traced ‘Enterprise’s Chr. 7 resistance allele to its progenitor, ‘Cox’s Orange Pippin’. Additionally, van de Weg et al. [16] reported putative epistatic QTLs on Chromosomes (Chrs.) 8 and 13 that demonstrated interactions with the Chr. 7 QTL. In a recent study, QTLs on Chrs. 6, 7, and 15 were detected and characterized across a pedigree-connected apple breeding germplasm set (n = 314 offspring) that represented 27 IBPs [43]. The Chrs. 6 and 15 QTLs detected by Kostick et al. [43] colocalized with previously reported QTLs [13,39]. Together, the Chrs. 6, 7, and 15 QTLs detected by Kostick et al. [43] explained approximately 28% of variation for SLB BLUPs [43], less than the upper range of average heritability estimates (0.22–0.49) for SLB data in this germplasm set [33]. The remaining unexplained heritability is likely due to multiple undetected small effect, epistatic and/or more environmentally dependent QTLs, or uncharacterized QTLs, such as those on Chrs. 8 and 16 reported by Kostick et al. [43]. The Chr. 16 QTL was not stable between years and the large QTL confidence interval on Chr. 8 could indicate multiple QTLs [43].

The effects of haplotypes (alleles) that underlie the Chrs. 6, 7, and 15 QTLs were characterized by Kostick et al. [43], using the terms “reduced susceptibility’ and “increased susceptibility” to describe alleles significantly associated with low and high relative susceptibility, respectively, in the pedigree-connected apple breeding germplasm set. Most of the 51 fire blight QTL haplotypes (alleles) characterized did not have significant effects (i.e., neutral effect) while six alleles were significantly associated with reduced susceptibility and four alleles were significantly associated with increased susceptibility across the three stable QTLs [43]. Although reduced-susceptibility alleles do not correspond to complete host resistance, they might contribute to achieving durable resistance in the long-term.

## 7. Short-Term Strategies for Breeding Apple Cultivars with Reduced Susceptibility to Fire Blight

### 7.1. Published Phenotypic and Fire Blight QTL Allele Information to Inform Parental Selection

**Figure 2:**
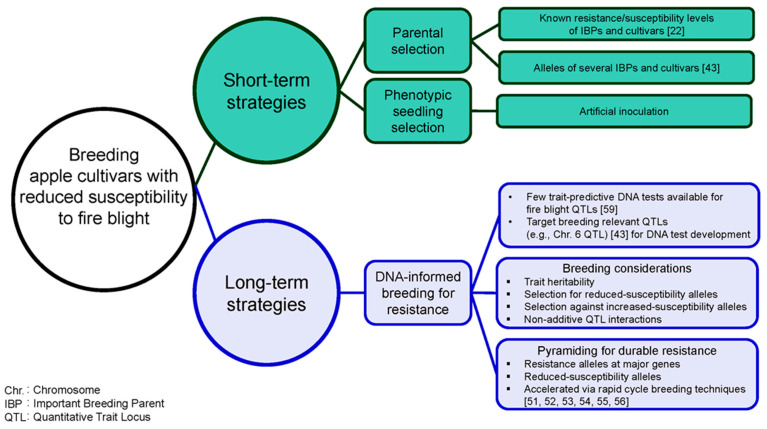
Short- and long-term strategies for breeding apple cultivars with durable resistance to fire blight. In the short-term, published phenotypic and genetic information could guide parental selection and phenotypic seedling selection could be used to cull highly susceptible individuals. DNA-informed breeding for resistance to fire blight is a long-term breeding strategy.

Recently published resistance/susceptibility classifications [22] and fire blight QTL allele information [43] of several IBPs and cultivars (n = 91) could inform selection of breeding parents (Figure 2). If low susceptibility to fire blight is desired in the next generation, breeders should consider avoiding IBPs that have zero reduced-susceptibility and/or multiple increased-susceptibility alleles at reported QTLs (e.g., Ginger Gold, Granny Smith, Minnewashta, Pinova, Sansa, and Sunrise). Kostick et al. [43] observed that higher numbers of reduced-susceptibility alleles across QTLs were generally associated with lower susceptibility responses, although interactions among QTLs were not purely additive. Selecting IBPs that are homozygous for reduced-susceptibility alleles at a given QTL and/or have multiple reduced-susceptibility alleles across relevant fire blight QTLs as parents (e.g., ‘Enterprise’) could be an effective breeding approach to developing improved breeding parents and/or cultivars with reduced susceptibility to fire blight [43].

### 7.2. Phenotypic Seedling Selection to Develop Populations with Low Susceptibility to Fire Blight

Artificial inoculation of seedlings derived from crosses targeting low susceptibility could be used to cull highly susceptible seedlings (Figure 2). Artificial inoculation of seedlings under greenhouse conditions often overestimates susceptibility, and thus might not be predictive of field performance [17]. However, phenotypic seedling selection is a cost-effective and relatively efficient approach used in apple breeding programs (e.g., WABP) to develop breeding populations with low susceptibility to fire blight.

## 8. DNA-Informed Breeding for Resistance to Fire Blight, a Long-Term Strategy

Use of DNA information in breeding decisions (i.e., DNA-informed breeding), which has become routine for several traits (e.g., resistance to apple scab, and malic acid content) in apple [58], would enable more efficient and accurate breeding for resistance to fire blight (Figure 2). Reduced-susceptibility alleles have been identified in multiple highly to moderately susceptible cultivars and IBPs such as Cox’s Orange Pippin, Gala, Hudson, Jonathan, Melrose, Northern Spy, and Yellow Newton [16,43]. Because of segregation at multiple additive and/or epistatic QTLs, parent resistance/susceptibility levels might not be indicative of offspring performance. Therefore, DNA-informed breeding is likely a more effective long-term approach compared to relying solely on phenotypic selection.

### 8.1. Few Trait-Predictive DNA Tests for Fire Blight QTLs Are Available

Although over 40 QTLs associated with resistance/susceptibility to fire blight have been reported [12,13,14,16,19,20,21,36,37,38,39,40,41,42,43], routine DNA-informed breeding for resistance is currently limited by the relatively few trait-predictive DNA tests available for fire blight QTLs relevant to apple breeding germplasm [7,59]. Simple sequence repeat (SSR), sequence characterized amplified region (SCAR), and SNP markers associated with various fire blight QTLs (e.g., Chrs. 3, 7, and 12) have been reported for use in DNA-informed breeding, e.g., [19,60,61,62]. Kellerhals et al. [7] described how markers associated with the Chrs. 3 and 7 QTLs are being used in a rapid cycle breeding program to efficiently select offspring that have inherited resistance alleles. QTLs recently detected in breeding relevant germplasm are additional useful targets for DNA test development, e.g., [43].

### 8.2. Chromosome 6 Fire Blight QTL Should Be Targeted for DNA Test Development

A Chr. 6 QTL detected by Kostick et al. [43] (which colocalized with a Chr. 6 QTL detected by Khan et al. [39]), segregated in families derived from ‘Honeycrisp’, an important U.S. cultivar and parent of several emerging cultivars including New York 1 (SnapDragon^®^ apple) and WA 38 (Cosmic Crisp^®^ apple) [63,64,65]. This Chr. 6 QTL should be targeted for DNA test development due to the breeding relevance of ‘Honeycrisp’ and the identification of a rare reduced-susceptibility allele that traced back to ‘Honeycrisp’s highly resistant progenitor, ‘Frostbite’ [43]. ‘Honeycrisp’s other Chr. 6 QTL allele was associated with increased susceptibility [43]. Selection for reduced-susceptibility and against increased-susceptibility alleles in parents or seedling populations derived from ‘Honeycrisp’ or related individuals might be an effective approach for developing breeding populations with low susceptibility to fire blight. The reduced-susceptibility allele derived from ‘Honeycrisp’ could be combined with resistance or reduced-susceptibility alleles at other fire blight QTLs for more durable resistance in the long-term.

### 8.3. Selection Against Susceptibility

Plant *R* genes, which often encode intracellular nucleotide-binding leucine-rich-repeat (NB-LRR) proteins, typically recognize specific pathogen-derived effectors (avirulence proteins) to induce host defense responses (effector-triggered immunity) [66]. Candidate fire blight *R* genes (CC-NBS-LRR, serine/threonine kinase, NBS-LRR, receptor-like kinase genes) have been reported for the Chrs. 3, 10, and 12 QTLs in *M. robusta* 5, *M. fusca*, and ‘Evereste’, respectively [44,45,46,47]. In contrast to *R* genes, susceptibility (*S*) genes are host genes that facilitate infection and compatible host-pathogen interactions [66]. Mutation or loss of an *S* gene in the host could limit a pathogen’s ability to cause disease leading to a host plant with lower susceptibility [66].

Van Schie and Takken [66] reviewed possible applications of targeting *S* genes when breeding for disease resistance in plants. In a recent study, Tegtmeier et al. [67] investigated genomic diversity of candidate fire blight *S* genes (*HIPM* and *DIPM* genes) in *Malus* germplasm. Tegtmeier et al. [67] argued that targeting *S* genes might be a more durable approach to breeding apple cultivars with low susceptibility to fire blight. To identify candidate genes for the Chrs. 6, 7, and 15 QTLs, Kostick et al. [43] examined functional annotations of genes within the QTL intervals. Annotations for 74 (18%), 81 (22%), and 90 (22%) genes in the Chrs. 6, 7, and 15 QTL intervals, respectively, indicated involvement in responses to disease and biotic stresses [43]. However, the causal genes underlying most reported fire blight QTLs are unknown.

Effects of QTL alleles are defined as phenotypic contrasts regardless of the causal genes (e.g., *R* genes, *S* genes) underlying QTL intervals. Kostick et al. [43] determined allelic effects by comparing presence vs. absence of a given allele using analysis of variance, defining alleles that had significantly higher and lower mean susceptibility levels (i.e., SLB BLUPs) as increased- and reduced-susceptibility (i.e., high and low relative susceptibility) alleles, respectively. Although most QTL mapping studies for disease-related traits in plants focus on characterizing alleles associated with resistance or reduced susceptibility (i.e., low relative susceptibility), knowledge of increased-susceptibility alleles that segregate in breeding germplasm could be used to inform parent selection and aid in culling decisions (e.g., DNA-informed seedling selection) once DNA tests for breeding relevant QTLs have been developed. For example, an increased-susceptibility allele at the Chr. 15 QTL detected by Kostick et al. [43] was prevalent among IBPs and might partially explain high to moderate susceptibility levels of several cultivars (e.g., Akane, Elstar, Gala, and Sweet Sixteen).

### 8.4. Non-Additive Interactions at and among Fire Blight QTLs

Non-additive interactions at and among fire blight QTLs have been reported [16,43]. Kostick et al. [43] reported that lower susceptibility levels were observed for offspring with higher numbers of reduced-susceptibility alleles across the Chrs. 6, 7, and 15 QTLs. However, additional reduced-susceptibility alleles did not always correspond to significantly lower susceptibility levels, indicating non-additive interactions at and across QTLs [43]. Both reduced- and increased-susceptibility alleles affect offspring responses to fire blight. Simultaneous selection for reduced- and against increased-susceptibility alleles might be an effective approach to developing breeding populations with low susceptibility to fire blight.

### 8.5. DNA-Informed Breeding to Achieve Durable Resistance

As multiple QTLs underlie variation for resistance/susceptibility to fire blight and QTL alleles characterized often only have moderate effects [43], breeders will need to pyramid resistance alleles at major genes derived from wild germplasm with multiple reduced-susceptibility alleles from elite (i.e., superior fruit quality) germplasm. Pyramiding of resistance/reduced-susceptibility alleles will enable (1) development of improved breeding parents enriched with favorable alleles; (2) achievement of desired resistance levels; and (3) development of cultivars with durable resistance to fire blight. Selecting individuals in which multiple favorable alleles have been combined is challenging when relying solely on phenotypic information [68]; therefore, trait-predictive DNA tests for relevant fire blight QTLs are needed for breeders to efficiently pyramid favorable alleles. Once DNA tests for relevant fire blight QTLs and fruit quality loci are available, rapid cycle breeding techniques [51,52,53,54,55,56] could be used to accelerate effective introgression and pyramiding of favorable alleles.

## 9. Conclusions: Durable Resistance to Fire Blight Could Be Efficiently Achieved through Breeding

Development of apple cultivars with durable resistance to fire blight and superior fruit quality could be efficiently achieved through DNA-informed breeding; however, progress is hampered by the few trait-predictive DNA tests that are available. In the short-term, published (1) phenotypic resistance/susceptibility information [22] and (2) reduced- and increased-susceptibility allele information for several IBPs and cultivars [43] could be applied immediately to inform selection of parents in apple breeding programs. Breeding-relevant QTLs that have been previously characterized could be targeted for development of DNA tests for breeders to pyramid favorable alleles and/or combine superior fruit quality with resistance to fire blight. Introgression and pyramiding of favorable alleles could be accelerated with rapid cycle breeding techniques.

## Data Availability

Not applicable.
